# Minimally invasive segmentectomy for non-small cell lung cancer (NSCLC): a comparative analysis of robotic and thoracoscopic approaches

**DOI:** 10.1186/s12893-025-03421-7

**Published:** 2025-12-19

**Authors:** Zeeshan Malik, Yanwu Zhou, Chunfang Zhang

**Affiliations:** Department of Thoracic surgery, Xiangya Hospital, Central South University, Kaifu District, Changsha, 410008 China

**Keywords:** Robot-assisted thoracoscopic surgery, Video-assisted thoracoscopic surgery, Segmentectomy, Lobectomy, Non-small cell lung cancer

## Abstract

**Background and objective:**

Anatomical segmentectomy is a lung-sparing surgical option for early-stage non-small cell lung cancer (NSCLC), especially in patients with small peripheral tumors or reduced pulmonary reserve. With increased detection via low-dose CT screening, the demand for segmentectomy is rising. This retrospective cohort study compared short-term outcomes, complications, and costs between robot-assisted segmentectomy (RAS) and video-assisted segmentectomy (VAS) in NSCLC patients.

**Methods:**

A sum of 603 patients with pathologically confirmed NSCLC who underwent segmentectomy at Xiangya Hospital between May 2020 and June 2024 were included: RAS (*n* = 302), VAS (*n* = 301). Both groups were comparable in demographics and baseline characteristics. Intraoperative variables, postoperative recovery indicators, lung function (pre- and postoperative), hemoglobin levels, arterial blood gases, and total cost were analyzed. Statistical significance was set at *p* < 0.05.

**Results:**

No 30-day mortality occurred in either group. RAS showed significantly shorter operation time (89.53 ± 26.60 min vs. 107.80 ± 43.92 min, *p* < 0.0001), faster intersegmental plane identification (6.10 ± 1.02 min vs. 14.07 ± 2.69 min, *p* < 0.0001), less blood loss (47.3 ± 39.7 ml vs. 57.3 ± 64.7 ml, *p* < 0.022), shorter hospital stay (7.9 ± 2.0 vs. 8.5 ± 3.6 days, *p* < 0.013), chest tube duration (3.3 ± 1.1 vs. 3.6 ± 1.3 days, *p* < 0.003), and POD1 drainage (257.6 ± 100.4 vs. 282.0 ± 118.4 ml, *p* < 0.006). RAS had fewer conversions (0.66% vs. 1.3%) and higher lymph node sampling (4.9 ± 0.1 vs. 4.1 ± 0.8, *p* < 0.0001). Minor (13.57% vs. 19.93%, *p* < 0.0387) and major (9.60% vs. 12.0%, *p* < 0.035) complications, postoperative pain (1.65% vs. 7.98%, *p* < 0.0002), cough, opioid (20% vs. 58%) and antitussive use (20% vs. 46.2%) were lower in RAS. Lung function and ABG values and haemoglobin were statistically similar. Total cost was significantly higher in RAS (9422.3 ± 3183.0 vs. 5741.4 ± 1223.2 USD, *p* < 0.0001).

**Conclusion:**

RAS offers better perioperative outcomes and lower morbidity than VAS but at significantly higher cost, requiring further strategies for cost optimization.

## Introduction

Lung segmentectomy is an anatomical resection that removes one or more bronchopulmonary segments while preserving healthy surrounding tissue. Unlike lobectomy, which involves resecting an entire lobe, segmentectomy targets smaller anatomical units, offering a lung-sparing alternative for early-stage non-small cell lung cancer (NSCLC) patients, particularly those with compromised pulmonary function [1]. Once reserved for benign lesions or patients unfit for lobectomy, segmentectomy is now widely accepted for tumors < 2 cm, especially those with ground-glass opacity (GGO) features or peripheral locations where safe margins can be ensured [2]. With advancements in imaging and surgical techniques, segmentectomy has become more precise and applicable to a broader population. Minimally invasive approaches, particularly video-assisted thoracoscopic surgery (VATS) and robot-assisted thoracoscopic surgery (RATS), have revolutionized thoracic surgery by reducing morbidity, postoperative pain, and recovery time compared to open thoracotomy [3, 4]. VATS, introduced in the early 1990 s, quickly gained popularity due to its benefits, but it has limitations, such as rigid instruments, two-dimensional visualization, and a steep learning curve, especially for complex anatomical segmentectomies [5]. RATS was developed to address these limitations. Systems like the da Vinci Surgical System offer three-dimensional, high-definition visualization, enhanced dexterity through wristed instruments, and improved ergonomics [6]. These features facilitate precise dissection and lymphadenectomy, making RATS particularly appealing for segmentectomy. However, RATS comes with high initial and ongoing costs, including expensive instruments and specialized training requirements, posing barriers in resource-limited settings [7]. Despite technical advantages, RATS and VATS remain comparable in many clinical outcomes, and there is no clear consensus on superiority. Some studies suggest RATS leads to better lymph node dissection, reduced blood loss, and lower conversion rates to open thoracotomy, while others report similar or even longer operative times [8–10]. Randomized trials like JCOG0802/WJOG4607L and CALGB 140,503 have confirmed the non-inferiority of segmentectomy to lobectomy in NSCLC ≤ 2 cm, solidifying its role in early-stage lung cancer [11, 12]. In China, increased adoption of low-dose CT screening has led to more early-stage diagnoses, encouraging the use of segmentectomy, often guided by advanced imaging like 3D-CT and AI-assisted planning [13, 14]. Recent findings suggest RATS may offer benefits such as lower blood loss, shorter chest tube duration, and better lymph node harvest, though VATS remains more accessible and less costly [15, 16].

Despite progress, there remains a lack of focused studies comparing robot-assisted segmentectomy (RAS) and video-assisted segmentectomy (VAS) segmentectomy specifically in patients with small pulmonary nodules (< 30 mm), particularly GGOs and minimally invasive adenocarcinomas. This study aims to address that gap by evaluating perioperative outcomes, complications, and cost in such patients, providing essential evidence to guide clinical decisions and policy-making.

## Methods

### Study design and patients

The Institutional Review Committee of Xiangya Hospital, Central South University, approved this retrospective study. Patients who underwent anatomical segmentectomy between May 2020 and June 2024 were categorized into robot-assisted segmentectomy (RAS, *n* = 302) and video-assisted segmentectomy (VAS, *n* = 301) groups. Eligible patients were aged 20–80 years with ground glass opacity (GGO) or semi-solid nodules ≤ 30 mm. Consolidation-to-Tumor Ratio (CTR) of the part-solid nodules was < 50% of the tumor diameter. Exclusion criteria included missing key outcome indicators, day-care surgery, combined segmentectomy and wedge resection, lobectomy, or purely solid nodules. All surgeries were conducted using standard anatomical segmentectomy, involving dissection and division of the segmental artery, vein, and bronchus. Preoperative evaluation included pulmonary function tests, ECG, echocardiography, blood gas analysis, CT scans, and in selected cases, PET-CT. All records were translated from Chinese to English for analysis.

### Surgical technique

**Fig. 1:**
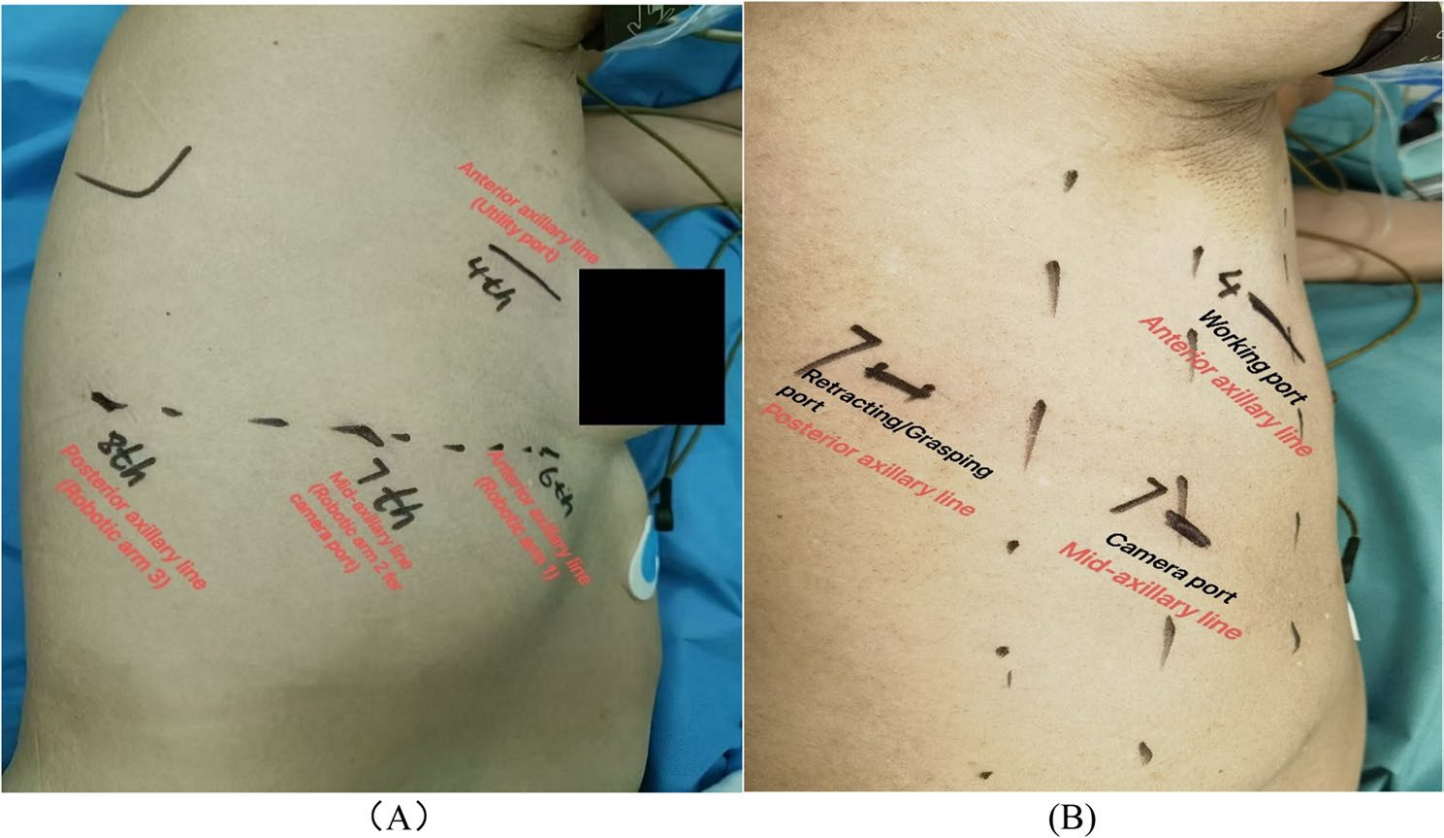
**A**. Patient surgical position for four arms Robot-assisted segmentectomy (RAS). **B**. Patient surgical position for three ports Video-assisted segmentectomy (VAS)

**Fig. 2:**
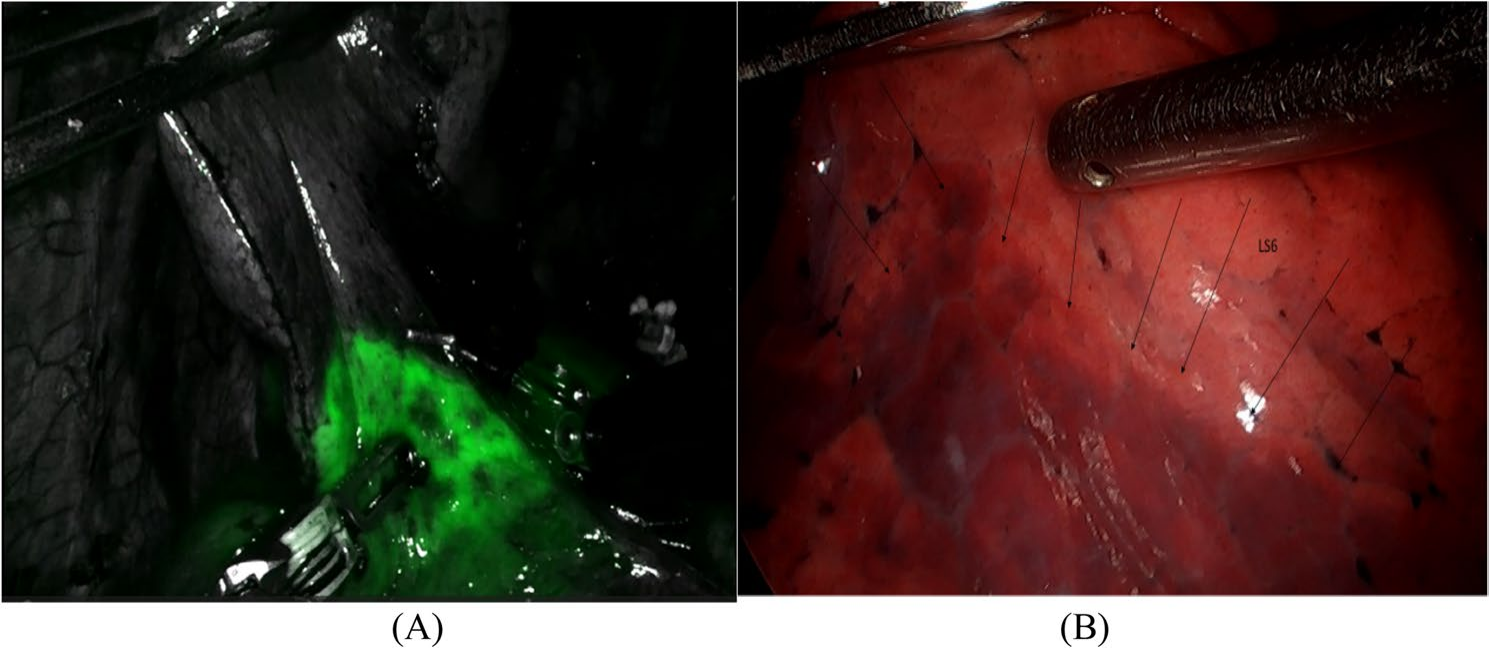
**A**. ICGF for the identification of the intersegmental plane of segment (LS1 + 2) by the arterial ligation method during RAS surgery. **B**. LS6 demarcation between the inflated and deflated areas during VAS surgery

Both RAS and VAS were performed under general anesthesia using double-lumen endotracheal intubation in the lateral decubitus position. RAS utilized the Da Vinci Xi system with three robotic ports (anterior axillary line 6th intercostal space (ICS), mid-axillary line 7th ICS and posterior axillary line 8th ICS) and one utility incision at 4th ICS, Fig. 1 (A). VAS was performed using a conventional three-port approach (two small incisions both at 7th ICS, mid and posterior axillary line respectively) with a working incision at the 4th ICS, Fig. 1 (B). Preoperative three-dimensional (3D) reconstruction imaging was performed for all patients in both groups to facilitate accurate visualization of the segmental veins, arteries, and bronchi. In RAS, intersegmental planes were delineated using indocyanine green (ICG) fluorescence imaging, Fig. 2 (A). In VAS, the inflation-deflation method was used, Fig. 2 (B). Segmental structures were divided using manual or electric staplers. Robotic staplers were not used. Intraoperative biopsy confirmed resection margins. Two 24-Fr chest tubes were placed for drainage. Conversion was defined as a switch to thoracotomy due to intraoperative difficulties.

### Outcome indicators

Perioperative outcomes included operative time, time from application of intersegmental plane identification to specimen submission for biopsy, number of lymph node dissection/sampling, blood loss, conversion rate, chest tube duration, postoperative day 1 drainage (POD1), and total hospital stay. Lung function was assessed preoperatively and one month postoperatively. Postoperative physiological indicators included hemoglobin, pCO₂, pO₂, and lactate levels. Pain and cough intensity were measured using the Numerical Pain Scale (NPS) and Numerical Rating Scale (NRS). Complications were classified as minor or major. Economic evaluation included detailed cost breakdowns.

### Statistical analysis

Continuous variables were compared using t-tests and expressed as mean ± SD; lactate values as mean ± SEM. Categorical variables were analysed using chi-square tests; one-way ANOVA was used for comparisons involving multiple groups. Statistical analysis was performed using GraphPad Prism 10.1.2. A *p*-value < 0.05 was considered significant.

## Results

### Demographic and basic characteristics of the patients

A total of 603 patients underwent anatomical segmentectomy for NSCLC, including 302 in the RAS group and 301 in the VAS group. Patients in the RAS group were generally older, and the VAS group had a higher proportion of benign or other lesions. No significant differences were noted in sex, smoking history, tumor size, pathological stage, or tumor location. Most patients had stage I disease, with adenocarcinoma as the predominant histologic type. No postoperative deaths occurred within 30 days in both groups. Table 1.

**Table 1:** Comparison of baseline demographic and clinical features between patients undergoing RAS and VAS segmentectomy

Variable	RAS (*n* = 302)	VAS (*n* = 301)	*p*-value
Sex, n (%)			
Male	123 (40.7)	103 (34.2)	0.183
Female	179 (59.3)	198 (65.8)	0.327
Smoking history, n (%)	163 (54.0)	159 (52.8)	0.806
Age, years (mean ± SD)	56.4 ± 10.2	53.8 ± 11.4	< 0.003
Tumor size, mm (mean ± SD)	13.8 ± 5.3	14.1 ± 5.2	0.639
Operation style, (n)			
Segmentectomy	302	301	0.999
Mortality (< 30 day)	0 (0)	0 (0)	
Pathological type, n (%)			< 0.0001
Adenocarcinoma	272 (90.1)	256 (85.0)	
Squamous cell carcinoma	2 (0.7)	3 (1.0)	
Benign/Others	28 (9.3)	42 (14.0)	
Pathological Staging, n (%)			0.414
I	241 (79.8)	227 (75.4)	
II	30 (9.9)	23 (7.6)	
III	3 (1.0)	9 (3.0)	
IV	0 (0)	0 (0)	
Tumor site, n (%)			0.993
Right upper lobe	99 (32.8)	91 (30.2)	
Right middle lobe	2 (0.66)	3 (0.99)	
Right lower lobe	64 (21.1)	59 (19.6)	
Left upper lobe	91 (30.1)	101 (33.5)	
Left lower lobe	46 (15.2)	47 (15.6)	

*RAS* Robot-assisted segmentectomy, *VAS* Video-assisted segmentectomy, *SD * standard deviation

### Comparison of surgical outcomes between RAS and VAS

Sum of 603 cases extracted from the database for analysis, comprising (*n* = 302) cases in RAS group and (*n* = 301) cases in the VAS group. All patients underwent segmentectomy. A surgical comparison between the two groups demonstrated that, there was no (< 30 day) mortality in both groups. Compared with the VAS group, the RAS group had significantly less duration of operation time (89.53 min ± 26.60 min vs. 107.80 ± 43.92 min, *p* < 0.0001), time difference from applications to obtain ISP to specimen submission (6.10 ± 1.02 min vs. 14.07 ± 2.69 min, *p* < 0.0001). Lower intraoperative blood loss (47.3 ± 39.7 ml vs. 57.3 ± 64.7 ml, *p* < 0.022), and a shorter duration of hospital stay (7.9 ± 2.0 days vs. 8.5 ± 3.6 days, *p* < 0.013). Additionally, the RAS group exhibited a shorter duration of chest tube placement and a smaller volume of chest tube drainage on postoperative day 1 (POD1). The average duration to chest tube extubation between two groups was (3.3 ± 1.1 days vs. 3.6 ± 1.3 days, *p* < 0.003). Similarly, the amount of chest tube drainage on POD1 was (257.6 ± 100.4 ml vs. 282.0 ± 118.4 ml, *p* < 0.006). The conversion rate to open thoracotomy was also lower in the RAS group, with only 0.66% (2/302) vs. 1.3% (4/301) in the VAS group. Regarding preoperative and postoperative haemoglobin levels, no significant differences were observed between the two groups, haemoglobin levels between two approaches before and after surgery were (130.6 ± 13.0, 122.9 ± 13.7) g/L for RAS and (130.8 ± 12.8, 121.8 ± 14.3) g/L for VAS, (*p* = 0.999). Moreover, we compared arterial blood gas (ABG) parameters between the two groups, including partial pressure of carbon dioxide (pCO₂), partial pressure of oxygen (pO₂), and lactate levels to observe the rapid changes after resection. All three parameters showed no statistically significant differences: pCO₂ (44.4 ± 6.3 mmHg vs. 44.2 ± 5.9 mmHg, *p* = 0.681), pO₂ (134.6 ± 49.4 mmHg vs. 139.6 ± 50.4 mmHg, *p* = 0.681), and lactate (1.8 ± 0.5 mmol/L vs. 1.3 ± 0.04 mmol/L, *p* = 0.422). Tables 2 and 3.

**Table 2:** Comparison of surgical outcomes between RAS and VAS segmentectomy in NSCLC patients

Variable	RAS (*n* = 302)	VAS (*n* = 301)	*p*-value
Convert to open surgery, n (%)	2 (0.66)	4 (1.3)	-
Intraoperative blood loss (mL)	47.30 ± 39.70	57.30 ± 64.70	< 0.022
Time difference from application of methods to obtain ISP to specimen submission (min)	6.10 ± 1.02	14.07 ± 2.69	< 0.0001
Operation time (min)	89.53 ± 26.60	107.80 ± 43.92	< 0.0001
Number of lymph node dissection/sampling stations (n)	4.9 ± 0.9	4.1 ± 0.8	< 0.0001
Chest tube duration (days)	3.30 ± 1.10	3.60 ± 1.30	< 0.003
Postoperative drainage POD1 (mL)	257.60 ± 100.40	282.00 ± 118.40	< 0.006
Total hospital stay (days)	7.90 ± 2.10	8.50 ± 3.50	< 0.013

*RAS* Robot-assisted segmentectomy, *VAS* Video-assisted segmentectomy, *POD1* Postoperative day one

**Table 3:** Post-operative comparison of haemoglobin and arterial blood gases (ABGs) parameters in RAS vs. VAS

Variable	RAS ± SED (Post-operative)	VAS ± SED (Post-operative)	*p*-value
Haemoglobin (g/L)	122.90 ± 13.70	121.80 ± 14.30	0.999
pCO2 (mmHg)	44.40 ± 6.30	44.20 ± 5.90	0.681
pO2 (mmHg)	134.60 ± 49.40	139.60 ± 50.40	0.681
Lactate (mmol/L)	1.80 ± 0.50	1.30 ± 0.04	0.422

*g/L* Grams per liter, *mmHg* Millimeters of mercury, *mmol/L* Millimoles per liter, *SED* standard error mean

### Comparison of post-operative minor complications in RAS and VAS, n (%)

In the RAS group, 41 out of 302 patients (13.57%) experienced minor complications, significantly fewer than the 60 out of 301 patients (19.93%) in the VAS group (*p* < 0.0387). Air leakage occurred in 10 RAS patients (3.31%) and 18 VAS patients (5.98%) but resolute within two days in both groups. Other complications in the RAS group included postoperative cough in 10 patients (3.31%), increased blood pressure in 7 (2.32%), postoperative pain in 5 (1.65%), low-grade fever in 5 (1.65%), and shortness of breath in 4 (1.32%). In the VAS group, the most frequent complication was pain in 24 patients (7.98%), followed by air leakage (5.98%), postoperative cough in 13 (4.31%), and increased blood pressure in 5 (1.66%). The higher incidence of pain in VAS was attributed to intercostal nerve injury due to a larger (12 mm) trocar used for the camera port. Greater cough severity was likely due to rigid instruments causing more airway and lung trauma. While the RAS group associated with less trauma to lung tissue due its clear 3D vision and more flexible arms.

### Interpretation of postoperative pain (NPS) and cough (NRS) between RAS and VAS

In the VAS group, 41.7% (10/24) of patients reported mild to moderate pain (NPS 3.4 on a 1–5 scale), while 58.3% (14/24) had moderate to severe pain (NPS 7.5 on a 6–10 scale), with 58.3% requiring opioids. In the RAS group, 80% (4/5) experienced mild to moderate pain (NPS 3.0), and 20% (1/5) had moderate to severe pain (NPS 6.0), with opioid use at 20%. For postoperative cough, 53.8% (7/13) of VAS patients reported mild to moderate symptoms (NRS 4.0), and 46.2% (6/13) had moderate to severe symptoms (NRS 6.6), while antitussive use was 46.2%. In the RAS group, 80% (8/10) had mild to moderate cough (NRS 3.3), and 20% (2/10) had moderate to severe symptoms (NRS 6.5), with 20% requiring antitussives. Tables 4 and 5.

**Table 4:** Assessment of pain severity by NPS score between RAS and VAS

NPS grading	Category	VAS Group (301)	RAS Group (302)	*p*-value
0-scale	No pain	277 (92.03%)	297 (98.3%)	
	Post-operative pain	24 (7.97%) patients	5 (1.65%) patients	< 0.0002
1–5 scale	Mild to Moderate Pain	10 patients (41.7%)	4 patients (80.0%)	
	– Average Pain Score	3.4	2.6	
	– Opioids Use	Occasional	Occasional	
6–10 scale	Moderate to Severe Pain	14 patients (58.3%)	1 patient (20.0%)	
	– Average Pain Score	7.5	6	
	–Opioids Use	14 patients (100%)	1 patient (20%)	
	Total Opioids Use (Moderate to Severe Pain)	14 patients (58.3%)	1 patient (20.0%)	

*NPS* Numerical Pain Scale, *RAS* robot-assisted segmentectomy, *VAS* video-assisted segmentectomy

**Table 5:** Assessment of cough severity by NRS score between RAS and VAS

NRS grading	Category	VAS Group (301)	RAS Group (302)	*p*-value
0-scale	No cough	288 (95.68%)	292 (96.68%)	
	Post-operative cough	13 (4.32%) patients	10 (3.32%) patients	0.665
1–5 scale	Mild to Moderate Cough	7 patients (53.8%)	8 patients (80.0%)	
	-Average NRS Score	4.0	3.3	
	-Antitussive Used	Suhuang (Occasionally)	Suhuang (Occasionally)	
6–10 scale	Moderate to Severe Cough	6 patients (46.2%)	2 patients (20.0%)	
	-Average NRS Score	6.6	6.5	
	- Antitussive Used	Codeine (All patients)	Codeine (All patients)	
	% of antitussive drug use (Moderate to Severe Cough)	(46.2%)	(20.0%)	

*NRS * Numerical Rating Scale, *VAS* video-assisted segmentectomy, *RAS* robot-assisted segmentectomy

### Comparison of post-operative major complications in RAS and VAS, n (%)

VAS group was associated with more number of major complications. Persistent air leakage (> 5 days) was the major complication in both groups. Chylothorax, pulmonary infection, and one case of > 200 mL bleeding occurred only in VAS. No aspiration pneumonia, bronchoalveolar fistula, or reoperations were reported in either group. Table 6.

**Table 6:** Incidence of major surgical complications in RAS and VAS

Complication	RAS (*n* = 302) *n* (%)	VAS (*n* = 301) *n* (%)	*p*-value
Persistent pulmonary leakage > 5 days	15 (4.96)	16 (5.31)	
Chylothorax	5 (1.65)	7 (2.32)	
Pulmonary infection	3 (0.99)	6 (1.99)	
Aspiration pneumonia	0 (0.00)	0 (0.00)	
Active bleeding > 200 ml	0 (0.00)	1 (0.33)	
Bronchoalveolar fistula	0 (0.00)	0 (0.00)	
Total	29 (9.60)	36 (12)	< 0.035

*RAS* = robot-assisted segmentectomy; *VAS* = video-assisted segmentectomy; *ml* = millilitres

### Lung function analysis

Based on the comparison of perioperative lung function between the RAS and VAS groups, no statistically significant differences were observed in any of the measured parameters before or one month after surgery (all p-values > 0.05). Both groups showed a mild decline in lung function postoperatively, which is expected after segmentectomy, but the changes were comparable between the groups. Parameters such as FEV1, FVC, FEV1/FVC, MVV, and peak VO₂ showed similar trends, indicating that the short-term impact on pulmonary function was equivalent between robot-assisted and video-assisted segmentectomy. Table 7.

**Table 7:** Comparison of preoperative and one-month postoperative pulmonary function between RAS and VAS segmentectomy groups

Variables	Lung function assessment time	RAS group (*n* = 302)	VAS group (*n* = 301)	*p*-value
FEV1/L	-Before surgery -After 1 month of surgery	2.26 ± 0.51 2.08 ± 0.48	2.23 ± 0.55 1.98 ± 0.45	0.695 0.108
FEV1 (predicted)%	-Before surgery -After 1 month of surgery	99.30 ± 6.51 94.18 ± 6.16	99.74 ± 11.50 93.60 ± 3.70	0.742 0.440
FVC/L	-Before surgery -After 1 month of surgery	2.52 ± 0.62 2.32 ± 0.59	2.49 ± 0.63 2.18 ± 0.30	0.935 0.060
FVC (predicted)%	-Before surgery -After 1 month of surgery	109.40 ± 10.99 98.77 ± 10.50	108.20 ± 9.43 96.20 ± 8.70	0.394 0.070
FEV1/FVC%	-Before surgery -After 1 month of surgery	93.08 ± 20.22 91.36 ± 23.66	97.33 ± 10.66 88.69 ± 10.41	0.076 0.320
MVV L/min	-Before surgery -After 1 month of surgery	87.89 ± 19.28 86.94 ± 18.11	87.04 ± 19.09 82.46 ± 16.96	0.754 0.081
Peak VO2/ml/kg/min	-Before surgery -After 1 month of surgery	22.22 ± 1.94 19.85 ± 1.82	21.98 ± 1.89 19.36 ± 1.63	0.383 0.057

*FEV1/* forced expiratory volume in 1 s (liter),* FEV1 (predicted) %* forced expiratory volume in 1 s (percentage of predicted value), *FVC/L* forced vital capacity per liter, *FVC (predicted)%* forced vital capacity (percentage of predicted value), *FEV1/FVC%* ratio of forced expiratory volume in 1 s to forced vital capacity (expressed as a percentage), *MVV L/min* maximum voluntary ventilation (liter per minute),* Peak VO2/ml/kg/min * peak oxygen consumption (millilitres per kilogram per minute)

#### Cost comparison between RAS and VAS techniques

A detailed cost comparison in USD across six categories showed that medicine and hospital stay costs were significantly lower in the RAS group, while surgical procedure, surgical materials, and blood testing expenses were higher. No significant difference was observed in laboratory investigation costs. Despite some category-specific savings, the overall expenditure was significantly higher in the RAS group, averaging 9422.3 ± 3183.0 USD vs. 5741.4 ± 1223.2 USD in the VAS group (*p* < 0.0001), a 64% increase. Excluding surgery and material costs, surprisingly VAS costs were 8.3% higher. However, surgery and material costs in RAS were nearly double those in VAS, a significant decrease in surgery cost and surgical material cost are in need to achieve comparable cost-effectiveness. Table 8.

**Table 8:** Cost analysis between RAS and VAS techniques

Cost Category	RAS (Mean ± SD, USD)	VAS (Mean ± SD, USD)	*p*-value
Medicine	946.2 ± 314.4	1113.3 ± 362.7	< 0.0001
Hospital Stay	333.4 ± 80.5	397.9 ± 414.6	< 0.008
Laboratory Tests	344.0 ± 157.5	366.0 ± 171.2	0.101
Blood Tests	926.8 ± 218.6	885.0 ± 211.1	< 0.017
Surgery	3550.0 ± 804.9	903.2 ± 263.7	< 0.0001
Surgical Materials	3341.3 ± 2729.6	2075.8 ± 623.4	< 0.0001
Total	9422.3 ± 3183.0	5741.4 ± 1223.2	< 0.0001

*RAS* robot-assisted segmentectomy, *VAS* video-assisted segmentectomy, *US$* United States dollar, *SD* standard deviation

## Discussion

As lung segmentectomy becomes more widely adopted for treating small, early-stage non-small cell lung cancers (NSCLC) and benign pulmonary nodules, selecting the optimal surgical approach between RAS and VAS has become increasingly significant in clinical decision-making [17]. VAS has long been recognized for reducing surgical trauma, complications, and hospital stays compared to open thoracotomy [18]. However, the introduction of robotic platforms promises to enhance these advantages further by offering three-dimensional visualization, wristed instruments, and superior ergonomics. In the United States, robotic lung resections now account for up to 25% of all anatomical lung resections and have surpassed VATS in some centers [19].

RAS technology allows for greater precision in dissecting segmental bronchi and vessels, which is particularly valuable in segmentectomy. Conventional thoracoscopic studies have reported better outcomes with RAS in both segmentectomy and lobectomy, highlighting its potential superiority [20]. Our study contributes to this growing evidence by providing a direct comparison of RAS and VAS segmentectomy, with a particular focus on intraoperative techniques, postoperative complications, and cost-effectiveness.

One distinguishing feature of the RAS approach in our study was the application of indocyanine green fluorescence (ICGF) for identifying intersegmental planes. Following ligation of the segmental artery, ICG was administered intravenously to accurately delineate the target segment using fluorescence guidance [21]. This technique offered clearer and faster visualization compared to the inflation–deflation method used in VAS, which was often less precise and more time-consuming [22]. We observed a significant reduction in the time from anatomical plane identification to specimen submission in RAS (6.10 ± 1.02 min) compared to VAS (14.07 ± 2.69 min), with a mean time difference of nearly 8 min (*p* < 0.0001). This efficiency contributed to the shorter operative time in RAS (89.53 min vs. 107.80 min, *p* < 0.0001). These positive outcomes also support the inclusion of slightly older patients in the RAS group. Rather than being a limitation, this age difference may underscore a key clinical advantage of RAS, its maintained efficacy and safety in older patients without increased complications. This suggests broader applicability of robotic surgery across age groups, warranting further investigation in age-stratified studies. In addition to time efficiency, robotic instrumentation improved nodal dissection due to enhanced articulation and 3D magnified vision, enabling surgeons to identify and preserve vital structures with greater precision [23]. In contrast, VAS was hindered by rigid instruments and limited mobility, often complicating lymphadenectomy, particularly during mediastinal dissection [24]. This difference was reflected in our findings, where chylothorax occurred more frequently in VAS (2.32%) than in RAS (1.65%). The perioperative outcomes in our study clearly favored RAS. Operation time, number of lymph nodes dissected, first-day drainage volume (POD1), chest tube duration, and intraoperative blood loss were all significantly better in the RAS group. These findings differ from previous studies, such as one conducted in mainland China, which reported no significant differences in these parameters [25]. Notably, hospital stay in our RAS group was significantly shorter, unlike other reports where robotic surgery did not reduce hospital duration [26]. Drainage volume on the first postoperative day in RAS approached the ideal 200 mL threshold considered safe for chest tube removal [27]. This may explain the reduced readmission rate in RAS patients due to fluid accumulation. A recent comparative study in 2025 with smaller sample size reported longer operative times and a 9% conversion rate to open surgery in the RAS group, compared to only 0.6% in our larger sample. Additionally, that study noted longer hospital stays in robotic cases (8.8 vs. 7.90 days), although lymph node sampling outcomes remained comparable [28].

We categorized postoperative complications into minor and major. Minor complications, such as cough, pain, and short-term air leakages that did not delay discharge, occurred less frequently in RAS (13.57%) than in VAS (19.93%). Air leakage was the most frequent, though none exceeded two days. Importantly, RAS patients experienced significantly less postoperative pain. On postoperative day one, 24 patients in the VAS group reported pain, compared to only 5 in the RAS group (*p* < 0.0002). Among VAS patients, 41.7% experienced mild-to-moderate pain (mean-NPS 3.4), and 58.3% reported moderate-to-severe pain (mean-NPS 7.5). In contrast, 80% of RAS patients reported only mild-to-moderate pain (mean-NPS 2.6), and 20% had moderate-to-severe pain (mean-NPS 6.0). This difference was likely due to intercostal nerve irritation from the larger 12-mm camera port used in VAS compared to the standard 8-mm trocars in RAS. Our results are consistent with a 2024 study, that demonstrated lower pain levels in robotic surgery [29]. Opioid use was significantly higher in the VAS group (58.3%) compared to RAS (20%). However, most patients were opioid-free by discharge, higher opioid use may impede respiratory recovery [30]. Cough severity, as measured by the NRS scale, did not differ statistically (*p* = 0.665), but 46.2% of VAS patients required antitussive medication versus only 20% in RAS. These findings suggest that RAS leads to better postoperative symptom control, contributing to faster mobilization and earlier discharge (*p* < 0.013). Major complications were also lower in RAS (9.60%) compared to VAS (12.0%, *p* < 0.035), particularly with respect to prolonged air leakage (>5 days), chylothorax, and postoperative pulmonary infections. Our findings align with a recent study that also reported significantly fewer pulmonary infections in RAS (*p* < 0.02) [31].

Pulmonary function outcomes remained statistically comparable between groups. FEV1, FVC, FEV1/FVC, FEV1/FVC, MVV, and peak Vo2, all the parameters were non-significant at one day before surgery and one month after surgery between two groups. These results align with earlier studies suggesting that segmentectomy offers better long-term pulmonary function preservation than lobectomy.

Despite the many clinical advantages of RAS, cost remains a critical concern. Our analysis showed that the mean total cost in the RAS group was significantly higher than in the VAS group ($9422.3 ± 3183.0 vs. $5741.4 ± 1223.2, *p* < 0.0001). The cost of the surgical procedure and materials in RAS were nearly twice that of VAS (surgery: $3550.0 vs. $903.2; materials: $3341.3 vs. $2075.8, both *p* < 0.0001). Even though medication and hospital stay costs were slightly lower in RAS, they were insufficient to offset the total cost difference. Interestingly, when surgery-related costs were excluded, the cost in remaining categories were 8.3% higher in the VAS group, likely due to greater use of opioids and antitussives. A French study in 2024 proposed that complication reduction, ICU avoidance, and discounts could reduce robotic surgery costs [32], our study already demonstrates low complication rates and minimal ICU transfers in the RAS group. We believe that future cost reductions may be achievable through encouraging local manufacturing of robots and expanded insurance coverage [33].

Institutional factors such as case volume and infrastructure also impact surgical outcomes and cost-effectiveness. High-volume centers are more likely to absorb the upfront costs of robotic platforms due to higher procedural efficiency and better outcomes [34]. Furthermore, the RAS learning curve is shorter than that of VAS, where surgeons may require 50 to 60 cases to achieve proficiency [35]. A key strength of our study is its large sample size, which enhances statistical power and improves the generalizability of our findings. Rigorous data collection through operative notes and cost databases ensured minimal information bias.

### Limitation

A retrospective, single-center design limits broader applicability, and the absence of long-term oncologic outcomes such as recurrence and survival is a limitation. While some ongoing studies continue to support the oncologic efficacy of segmentectomy [36], future randomized controlled trials comparing RAS and VAS directly are essential. Furthermore, the involvement of patient data from three different surgical teams may introduce potential selection and surgeon-related biases.

## Conclusion

This comparative study of RAS and VAS in NSCLC patients revealed that, while both achieved complete resection with no 30-day mortality, RAS demonstrated superior short-term outcomes, including shorter operation time, less blood loss, faster recovery, fewer complications, and better lymph node dissection. Postoperative pain, cough, and major complications were less frequent in the RAS group. Pulmonary function outcomes were similar in both groups demonstrating comparable results. However, RAS incurred significantly higher overall costs, primarily due to surgical and material expenses, highlighting the need to balance clinical benefits with financial considerations in surgical planning.

## Data Availability

The datasets generated and analysed during the current study are available from the corresponding author on reasonable request.
